# Mechanisms of Obesity-Related Kidney Disease: From Adipose Depot Biology to the Chymase–Aldosterone and Ghrelin–Leptin Axes

**DOI:** 10.3390/biom16081155

**Published:** 2026-08-08

**Authors:** Hsuan-Chu Hsu, Li-Jane Shih, Yi-Chou Hou, Kuo-Cheng Lu

**Affiliations:** 1 Division of Nephrology, Department of Internal Medicine, Yangming Branch, Taipei City Hospital, Taipei 111024, Taiwan; dau87@tpech.gov.tw; 2 College of Medicine, National Yang Ming Chiao Tung University, Taipei 112304, Taiwan; 3 Department of Medical Laboratory, Taoyuan Armed Forces General Hospital, Taoyuan City 325208, Taiwan; genelab@aftygh.gov.tw; 4 Graduate Institute of Medical Science, National Defense Medical University, Taipei 11201, Taiwan; 5 School of Medicine, College of Medicine, Fu Jen Catholic University, New Taipei City 242062, Taiwan; 127097@mail.fju.edu.tw; 6 Division of Nephrology, Department of Internal Medicine, Cardinal Tien Hospital, School of Medicine, College of Medicine, Fu Jen Catholic University, New Taipei City 24205, Taiwan; 7 Division of Nephrology, Department of Medicine, Taipei Tzu Chi Hospital, Buddhist Tzu Chi Medical Foundation, New Taipei City 231405, Taiwan; 8 Division of Nephrology, Department of Internal Medicine, Fu Jen Catholic University Hospital, New Taipei City 24352, Taiwan

**Keywords:** adipokines, aldosterone synthase inhibitors, chronic kidney disease, chymase, ghrelin, glomerular hyperfiltration, GLP-1 receptor agonists, lipotoxicity, metabolic syndrome, obesity, obesity-related glomerulopathy, renin-angiotensin-aldosterone system

## Abstract

Obesity is an increasingly important and modifiable driver of chronic kidney disease (CKD), with effects that extend well beyond its associations with type 2 diabetes, hypertension, and dyslipidemia. To synthesize the evidence that excess adiposity is a causal and modifiable determinant of kidney disease, and to examine how specific adipose depots injure the glomerulus and the tubulointerstitium, and then map these mechanisms onto established and emerging therapies. Throughout, obesity-related kidney disease (ORKD) denotes the full spectrum of diposity-driven renal injury, whereas obesity-related glomerulopathy (ORG) is reserved for the biopsy-defined glomerular lesion. Central, visceral, perirenal and renal-sinus adiposity act first through structural and haemodynamic mechanisms, promoting glomerular hyperfiltration, mechanical renal compression and activation of the adipose-derived renin–angiotensin–aldosterone system (RAAS). In parallel, these depots drive cellular and metabolic injury through lipotoxicity, adipokine imbalance, sterile inflammation, oxidative stress, gut dysbiosis, mitochondrial dysfunction, epigenetic remodelling and cellular senescence. Ectopic lipid accumulation within the renal parenchyma—fatty kidney—offers a unifying description of these changes and is most marked in type 2 diabetes mellitus. These interacting processes converge on podocyte stress, tubular metabolic failure, endothelial dysfunction and interstitial fibrosis, producing a phenotypic continuum that ranges from early albuminuria to obesity-related glomerulopathy and progressive CKD. Within the RAAS limb we highlight two comparatively underappreciated, adiposity-linked routes to injury: adipocyte-derived leptin directly upregulates adrenal aldosterone synthase (CYP11B2), and mast-cell chymase generates angiotensin II independently of angiotensin-converting enzyme, together reinforcing aldosterone- and angiotensin II–mediated damage that conventional RAAS blockade only partially interrupts. We further consider the counter-regulatory ghrelin–leptin axis, in which the suppression of ghrelin that accompanies obesity may withdraw an antioxidant, anti-inflammatory and podocyte-protective signal precisely as leptin-driven glomerular injury intensifies, positioning ghrelin as a plausible modulator and candidate biomarker of obesity-related kidney injury. We also examine how obesity complicates renal risk assessment, drug dosing, dialysis delivery and transplant access. Emerging, mechanism-matched therapies—SGLT2 inhibitors, GLP-1 receptor agonists, finerenone, structured lifestyle intervention, metabolic-bariatric surgery and, most recently, aldosterone synthase inhibitors that suppress the chymase- and leptin-driven aldosterone escaping receptor blockade—now enable a precision cardiovascular-kidney-metabolic framework that aligns adipose-depot biology, biomarkers, histology and treatment response to guide mechanism-based care in ORKD.

## 1. Introduction

Obesity and chronic kidney disease (CKD) have become two of the defining public-health challenges of the twenty-first century, and their trajectories are increasingly intertwined [1]. In 2022, approximately 890 million adults were living with obesity, and the age-standardised prevalence of adult obesity has more than doubled since 1990 [2]. Over the same period, CKD has risen to affect more than a tenth of the global adult population and is now a leading contributor to cardiovascular morbidity, premature mortality and health-system expenditure [2,3]. The convergence of these epidemics carries particular clinical weight because obesity not only raises the risk of incident CKD but also accelerates the loss of kidney function and narrows access to optimal kidney replacement therapy [4,5,6].

Historically, the link between obesity and kidney disease was attributed to intermediary conditions—chiefly type 2 diabetes mellitus (T2DM), hypertension and dyslipidaemia [3,7,8]. Large cohort studies, meta-analyses and Mendelian randomisation analyses have since shown that excess adiposity confers renal risk independently of these mediators, reframing obesity from a downstream correlate to an upstream cause of kidney injury [8,9]. This shift has moved the field from epidemiological association towards a mechanistic model in which adipose tissue, kidney parenchyma, vascular endothelium, immune cells and the gut microbiome interact within a self-reinforcing network of metabolic injury [7,10]. It has also highlighted the limitations of body mass index (BMI), which cannot distinguish the visceral, perirenal and renal-sinus depots that most closely track intrarenal haemodynamic stress, inflammation and insulin resistance from metabolically inert subcutaneous fat or preserved muscle mass [11,12,13,14].

The prototypical structural lesion of this process is obesity-related glomerulopathy (ORG), defined by glomerulomegaly with or without a perihilar variant of focal segmental glomerulosclerosis (FSGS) and reflecting adaptive podocyte stress in response to sustained single-nephron hyperfiltration [15,16]. Yet ORG represents only one facet of a broader renal phenotype [7,16]. Excess adiposity also drives proximal-tubular lipotoxicity, mitochondrial stress, sterile inflammation, maladaptive repair and interstitial fibrosis, so that obesity-associated kidney disease is best understood as a multi-compartment, multi-hit disorder rather than a single glomerular entity [10].

A crucial unifying concept is fatty kidney disease, defined by ectopic accumulation of triglycerides and other lipid species within the renal parenchyma—particularly proximal tubular cells, podocytes and mesangial cells—together with expansion of renal sinus and perirenal fat depots. Imaging and autopsy studies indicate that renal lipid content increases with body mass index, visceral adiposity and insulin resistance, and that renal sinus fat is independently associated with hypertension and reduced kidney function [17]. Fatty kidney can be viewed as the renal counterpart of hepatic steatosis: in both, lipid is deposited in an organ not adapted for storage, emerging when energy delivery exceeds oxidative and storage capacity and accompanied by local insulin resistance, mitochondrial stress and inflammation. This parallel is substantive rather than merely descriptive, as steatotic liver disease and CKD often coexist and share common metabolic drivers.

This relationship is most pronounced in type 2 diabetes mellitus (T2DM). Insulin resistance increases free fatty acid flux from visceral adipose tissue while stimulating hepatic and renal de novo lipogenesis, exposing the diabetic kidney to lipid from both supply and synthesis. Renal steatosis is therefore more severe in T2DM than in obesity alone and commonly coexists with steatotic liver disease, which is linked to albuminuria and higher CKD incidence in diabetic populations. Fatty kidney may thus represent a shared substrate on which obesity- and diabetes-related injury converge, helping to explain residual kidney risk despite glycaemic and blood-pressure control in T2DM. Hepatology has adopted the term metabolic dysfunction-associated steatotic liver disease, but no analogous consensus nomenclature exists for the kidney, and current human data are predominantly cross-sectional and associative. Throughout this Review, obesity-related kidney disease (ORKD) denotes the full spectrum of adiposity-driven renal injury, encompassing both glomerular and tubulointerstitial compartments, whereas obesity-related glomerulopathy (ORG) is reserved for the specific biopsy-defined lesion of glomerulomegaly with or without perihilar focal segmental glomerulosclerosis.

Within this network, two features of the renin–angiotensin–aldosterone system (RAAS) are central to the present Review and remain comparatively underappreciated in obesity. First, adipocyte-derived leptin acts directly on adrenal zona glomerulosa cells to upregulate aldosterone synthase (CYP11B2), establishing a fat–adrenal axis that raises aldosterone independently of the classical regulators of its secretion [18,19]. Second, mast-cell chymase—abundant in both obese adipose tissue and the injured kidney—generates angiotensin II independently of angiotensin-converting enzyme, supplying a non-canonical route to angiotensin II– and aldosterone-mediated injury that conventional RAAS blockade does not fully interrupt [20,21]. Superimposed on these pro-injurious pathways is a counter-regulatory adipokine dimension: ghrelin, the principal orexigenic hormone and a functional counterpart to leptin, is suppressed in obesity, so that its antioxidant, anti-inflammatory and podocyte-protective actions may be withdrawn precisely as leptin-driven glomerular injury intensifies [22,23]. Together, the leptin–aldosterone, chymase–angiotensin II and ghrelin–leptin axes illustrate how adipose-depot biology is transmitted to the kidney through interlocking endocrine and paracrine signals.

**Figure 1:**
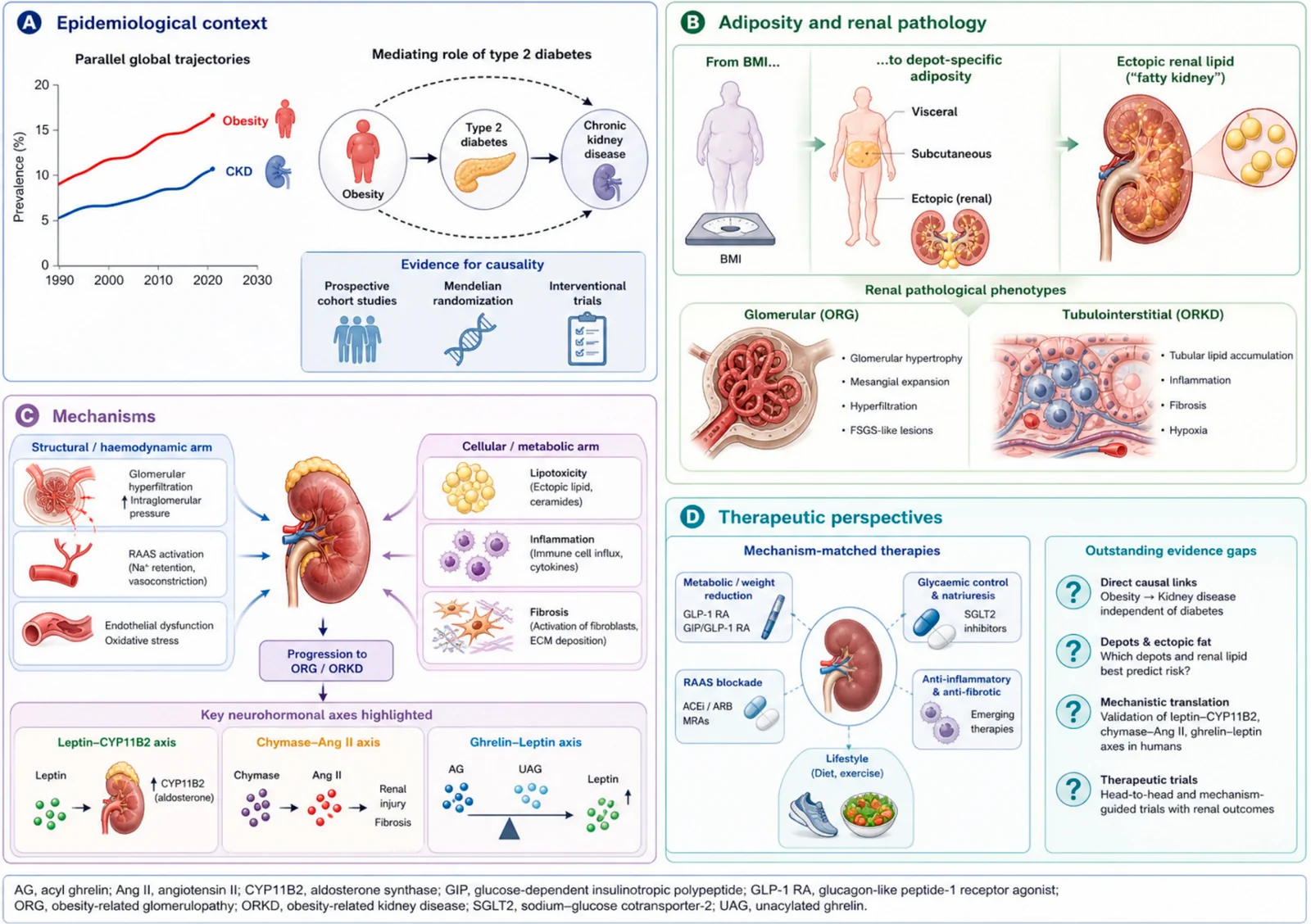
Conceptual overview and structure of this Review. (**A**) Epidemiological context: the parallel global trajectories of obesity and chronic kidney disease, the mediating role of type 2 diabetes and the evidence for causality. (**B**) Adiposity and renal pathology: the shift from body mass index to depot-specific exposure, ectopic renal lipid (fatty kidney), and the resulting glomerular and tubulointerstitial phenotypes. (**C**) Mechanisms: the structural/haemodynamic and cellular/metabolic arms, including the leptin–CYP11B2, chymase–angiotensin II and ghrelin–leptin axes emphasised here. (**D**) Therapeutic perspectives: mechanism-matched therapies and the outstanding evidence gaps. AG, acyl ghrelin; Ang II, angiotensin II; CYP11B2, aldosterone synthase; GIP, glucose-dependent insulinotropic polypeptide; GLP-1 RA, glucagon-like peptide-1 receptor agonist; ORG, obesity-related glomerulopathy; ORKD, obesity-related kidney disease; SGLT2, sodium–glucose cotransporter-2; UAG, unacylated ghrelin.

The therapeutic landscape has changed in parallel. Sodium–glucose cotransporter-2 (SGLT2) inhibitors and glucagon-like peptide-1 receptor agonists (GLP-1 RAs) have delivered clinically meaningful renal benefit in contemporary trials [24,25]; finerenone and metabolic-bariatric surgery offer complementary, kidney-relevant options in selected populations [26,27]; and aldosterone synthase inhibitors have emerged as an upstream strategy that suppresses aldosterone biosynthesis rather than blocking its receptor, capturing the chymase- and leptin-driven aldosterone that escapes conventional therapy [28,29,30]. In brief, this Review has three aims. First, to synthesise the epidemiological and clinicopathological evidence that excess adiposity is a causal and modifiable determinant of kidney disease, rather than merely a marker of the conditions that accompany it. Second, to examine how specific adipose depots transmit injury to the glomerulus and to the tubulointerstitium, with particular attention to three axes that remain under-recognised in this setting: leptin-driven aldosterone synthesis, chymase-derived angiotensin II, and the counter-regulatory ghrelin–leptin relationship. Third, to map these mechanisms onto established and emerging therapies, in order to define a precision cardiovascular-kidney-metabolic framework in which adipose-depot biology, validated biomarkers, histology and treatment response are aligned [4,10]. Figure 1 summarises the structure of this Review, from the epidemiological context and depot-specific pathology of ORKD, through the mechanistic axes that link adipose tissue to glomerular and tubulointerstitial injury, to the mechanism-matched therapies that now permit a precision cardiovascular-kidney-metabolic approach.

## 2. Epidemiology and Causal Inference

### 2.1. Obesity as a Driver of Incident CKD

Large cohorts consistently show a graded relationship between adiposity and kidney outcomes [4,31]. In the United States, higher BMI has been associated with a greater risk of end-stage kidney disease (ESKD), even after adjustment for diabetes and hypertension [5]. Similar patterns have been reported across Asian populations, where metabolic risk often appears at lower BMI thresholds than in European-ancestry cohorts [32,33]. Meta-analytic evidence indicates that overweight and obesity increase the risk of incident CKD in a dose-dependent manner, supporting a population-level contribution of high BMI to the global CKD burden [34,35].

The rise of type 2 diabetes mellitus (T2DM) is inseparable from this picture. Global T2DM prevalence has risen in close parallel with obesity, and diabetes is now the single leading cause of end-stage kidney disease worldwide; between roughly one third and two fifths of people with T2DM develop CKD during their lifetime. The burden falls disproportionately on Asian populations, in whom T2DM and kidney disease develop at lower BMI thresholds, consistent with the depot-specific risk described above [32,33,35]. T2DM is also presenting at progressively younger ages, so that recent birth cohorts accumulate longer cumulative metabolic exposure and diabetic kidney disease is increasingly encountered in younger adults. The rise in T2DM therefore accounts for a substantial part of the obesity-attributable CKD burden. It does not, however, account for all of it: obesity remains associated with incident CKD and end-stage kidney disease after adjustment for diabetes and hypertension [5], and ORG occurs in individuals with normal glucose tolerance [15,16]. Adiposity should thus be understood as acting both through diabetes and independently of it, and the mechanisms considered in Section 3 are relevant to both routes.

BMI is clinically useful but biologically incomplete [4,11]. Visceral, ectopic, and perirenal adiposity more closely track insulin resistance, inflammation, renal sinus fat expansion, and intrarenal haemodynamic stress than total body weight [12,13,14]. This distinction is particularly important in Asian populations, older adults, and patients with sarcopenic obesity, in whom BMI may underestimate metabolic risk [36,37]. Genetic-instrument studies further support a causal role for adiposity in lower estimated glomerular filtration rate (eGFR), albuminuria, and CKD risk, with stronger inference when adiposity is considered beyond BMI alone [8,9].

### 2.2. Progression of Established CKD

Obesity also modifies the clinical course of established CKD [38,39]. In CKD cohorts, metabolic syndrome and adverse adiposity-related risk profiles have been associated with higher risks of ESKD, while selected metabolic components are also associated with mortality [40]. Severe obesity appears particularly harmful because it combines glomerular hyperfiltration, renal compression, sympathetic activation, sleep apnoea, resistant hypertension and systemic inflammation [13,18,41]. Registry data further show that obesity is increasingly common among patients reaching dialysis or transplant evaluation, creating practical barriers or management considerations for vascular access, peritoneal dialysis, surgical eligibility and waitlisting [42,43,44].

The relationship is not uniform across all CKD stages [4]. Observational studies in dialysis populations have described an apparent “obesity paradox”, in which higher BMI is associated with lower mortality [45]. This finding requires cautious interpretation [45,46]. BMI does not distinguish adipose tissue from muscle mass, and reverse causation, protein-energy wasting, inflammation and survivor bias can make low BMI a marker of advanced illness rather than a protective comparator [45,46,47]. Studies incorporating body composition suggest that excess adiposity and low muscle mass have divergent prognostic implications, reinforcing the need to evaluate fat quality, fat distribution and muscle reserve rather than BMI alone [48].

### 2.3. Ageing, Sarcopenic Obesity and the Compounding of Renal Risk

Risk is not merely additive when obesity and T2DM occur in an ageing kidney. Nephron number declines with age through nephrosclerosis, so the hyperfiltration demand imposed by excess adiposity falls upon fewer functioning nephrons and produces greater single-nephron stress than the same exposure would in a younger kidney. Ageing, obesity and T2DM each additionally drive cellular senescence and a senescence-associated secretory phenotype in renal and adipose tissue, acting through the shared pathways of mitochondrial dysfunction, oxidative stress and chronic low-grade inflammation considered in Section 3.7; their combination is therefore plausibly synergistic rather than simply cumulative Because duration of diabetes is among the strongest predictors of diabetic kidney disease, the earlier onset of T2DM in recent cohorts means that an older adult today may carry several decades of metabolic exposure.

Sarcopenic obesity, the combination of excess adiposity with low muscle mass, becomes more prevalent with age and carries worse renal and mortality outcomes than either component alone [49,50]. It also creates a measurement paradox that causes risk to be systematically underrecognised in precisely the group in which it is highest: BMI underestimates adiposity when muscle mass is low, while creatinine-based eGFR simultaneously overestimates kidney function for the same reason. Cystatin C-based or combined creatinine–cystatin C equations, together with direct measurement of body composition, should therefore be preferred when assessing older adults with obesity [48,51,52].

Cardiovascular death competes with progression to kidney failure in older adults, so the absolute renal benefit of intervention may be smaller than in younger patients even where relative effects are preserved. Weight reduction must also be pursued differently, since unsupervised caloric restriction in sarcopenic obesity risks accelerating muscle loss and frailty and should be combined with resistance exercise and nutritional support [49]. The convergence of ageing, obesity and T2DM should therefore be regarded as a distinct high-risk phenotype rather than as the simple co-occurrence of three risk factors, and as one that is systematically underrepresented in the trials on which current recommendations rest.

## 3. Molecular and Physiological Mechanisms Linking Obesity to CKD

**Figure 2:**
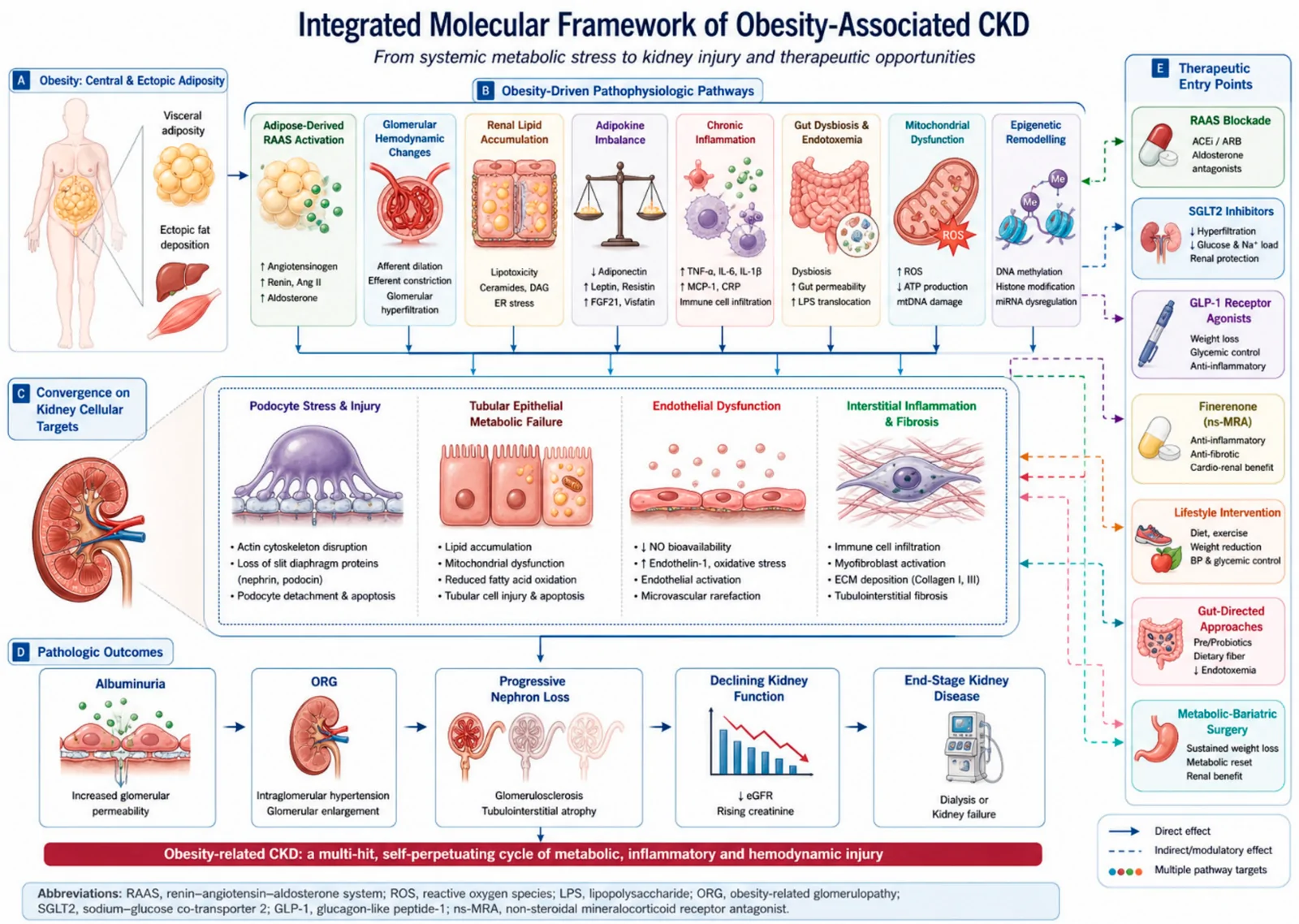
Integrated molecular framework of ORKD. (**A**) Central and ectopic adiposity promote (**B**) glomerular hyperfiltration, adipose-derived RAAS activation, renal lipid accumulation, adipokine imbalance, chronic inflammation, gut dysbiosis, mitochondrial injury and epigenetic remodelling. (**C**) These pathways converge on podocyte stress, tubular metabolic failure, endothelial dysfunction and interstitial fibrosis, (**D**) leading to albuminuria, ORG and progressive CKD. (**E**) Potential therapeutic entry points include RAAS blockade, SGLT2 inhibitors, GLP-1 receptor agonists, finerenone, lifestyle intervention, gut-directed approaches and metabolic-bariatric surgery.

Obesity-associated CKD is a multi-compartment, multi-hit disorder in which haemodynamic, endocrine, immune, metabolic, microbial, epigenetic and mitochondrial pathways converge on glomerular, tubular, endothelial and interstitial injury [4,10]. Hyperfiltration stresses podocytes; lipid excess disrupts mitochondrial fatty-acid oxidation; mitochondrial damage activates NLRP3 and cGAS-STING signalling; gut-derived toxins amplify oxidative stress; and adipokine imbalance sustains sympathetic, inflammatory and fibrotic responses [10,53]. This framework explains why kidney injury in obesity extends beyond obesity-related glomerulopathy and requires combined rather than single-pathway intervention (Table 1, Figure 2) [4,7].

### 3.1. Hemodynamic Load, Renal Compression, and RAAS Activation

Haemodynamic stress is among the earliest and most clinically tractable insults. Excess adiposity, renal sinus or perirenal fat expansion, sympathetic overactivity, insulin resistance and enhanced tubular sodium reabsorption increase renal plasma flow, single-nephron filtration and intraglomerular pressure [13,78]. Initially, adaptive hyperfiltration may preserve whole-kidney GFR, but sustained single-nephron hypertension stretches podocytes, promotes albuminuria and predisposes to segmental sclerosis [16]. Because albuminuria and hyperfiltration can precede irreversible scarring, early detection and timely pressure-lowering therapy are central to risk modification [79,80].

RAAS activation further amplifies fibrotic injury. Visceral adipocytes and stromal cells express angiotensinogen, angiotensin-converting enzymes and angiotensin receptors, creating a local endocrine-paracrine axis that can augment systemic RAAS tone [81]. Angiotensin II promotes efferent vasoconstriction, mesangial contraction and transforming growth factor-beta signalling, whereas aldosterone injures podocytes, tubular cells and endothelium through mineralocorticoid-receptor activation, oxidative stress and inflammation [56,82]. This biology underpins RAAS blockade and supports mineralocorticoid-receptor antagonism, including finerenone, in albuminuric metabolic kidney disease [57].

#### Perirenal and Renal-Sinus Adipose Tissue: Mechanical Versus Paracrine Mechanisms

Perirenal and renal-sinus fat merit separate consideration, because they act upon the kidney through two mechanistically distinct routes that are easily conflated under the single term “renal compression”. The first is purely mechanical. Expansion of these depots within the confined space bounded by the renal capsule and Gerota’s fascia raises interstitial hydrostatic pressure and compresses the renal vein, the vasa recta and the intrarenal lymphatics. Reduced medullary blood flow and slowed tubular flow increase sodium reabsorption, which lowers distal sodium delivery, suppresses tubuloglomerular feedback and thereby sustains afferent vasodilatation and hyperfiltration. This constitutes a physical, adipokine-independent route to intraglomerular hypertension—a form of intrarenal compartment physiology—and it is not corrected by pharmacological RAAS blockade [13,78].

The second route is paracrine and endocrine. Perirenal adipose tissue is anatomically contiguous with the renal hilum and is drained by vessels communicating with the renal circulation, so that free fatty acids, leptin, tumour necrosis factor-alpha, interleukin-6 and monocyte chemoattractant protein-1 may reach the renal parenchyma by short-range diffusion and local venous drainage rather than only through the systemic circulation. Perirenal fat also expresses angiotensinogen and angiotensin-converting enzyme and harbours chymase-positive mast cells, providing a local source of angiotensin II immediately adjacent to the kidney (Section 3.2), and it exhibits browning-related and sympathetic-innervation features that distinguish it from other visceral depots [20,81].

The distinction has practical consequences. The mechanical arm predicts a response to reduction of depot volume—through weight loss or metabolic-bariatric surgery—and is measurable by computed tomography or magnetic resonance imaging, whereas the paracrine arm predicts a response to anti-inflammatory, RAAS- and mineralocorticoid receptor-directed therapy and is better captured by circulating and tissue biomarkers. Perirenal fat thickness is accordingly proposed in Section 7 as a candidate stratifying variable for future trials.

### 3.2. Obesity-Driven Aldosterone and Chymase Pathways in Kidney Injury

In obesity, aldosterone is generated in excess through adipocyte-derived signals as well as through angiotensin II [18,56]. Leptin binds receptors co-expressed on CYP11B2-positive cells of the adrenal zona glomerulosa and dose-dependently increases aldosterone synthase expression and aldosterone secretion [18]. This fat–adrenal axis raises aldosterone independently of the classical regulators of its secretion and drives mineralocorticoid-receptor–mediated endothelial dysfunction and fibrosis [18]. This biology provides a rationale for suppressing aldosterone production itself rather than only blocking its receptor [83].

#### 3.2.1. Non-ACE Pathways of Angiotensin II Generation: Chymase Versus ACE

**Figure 3:**
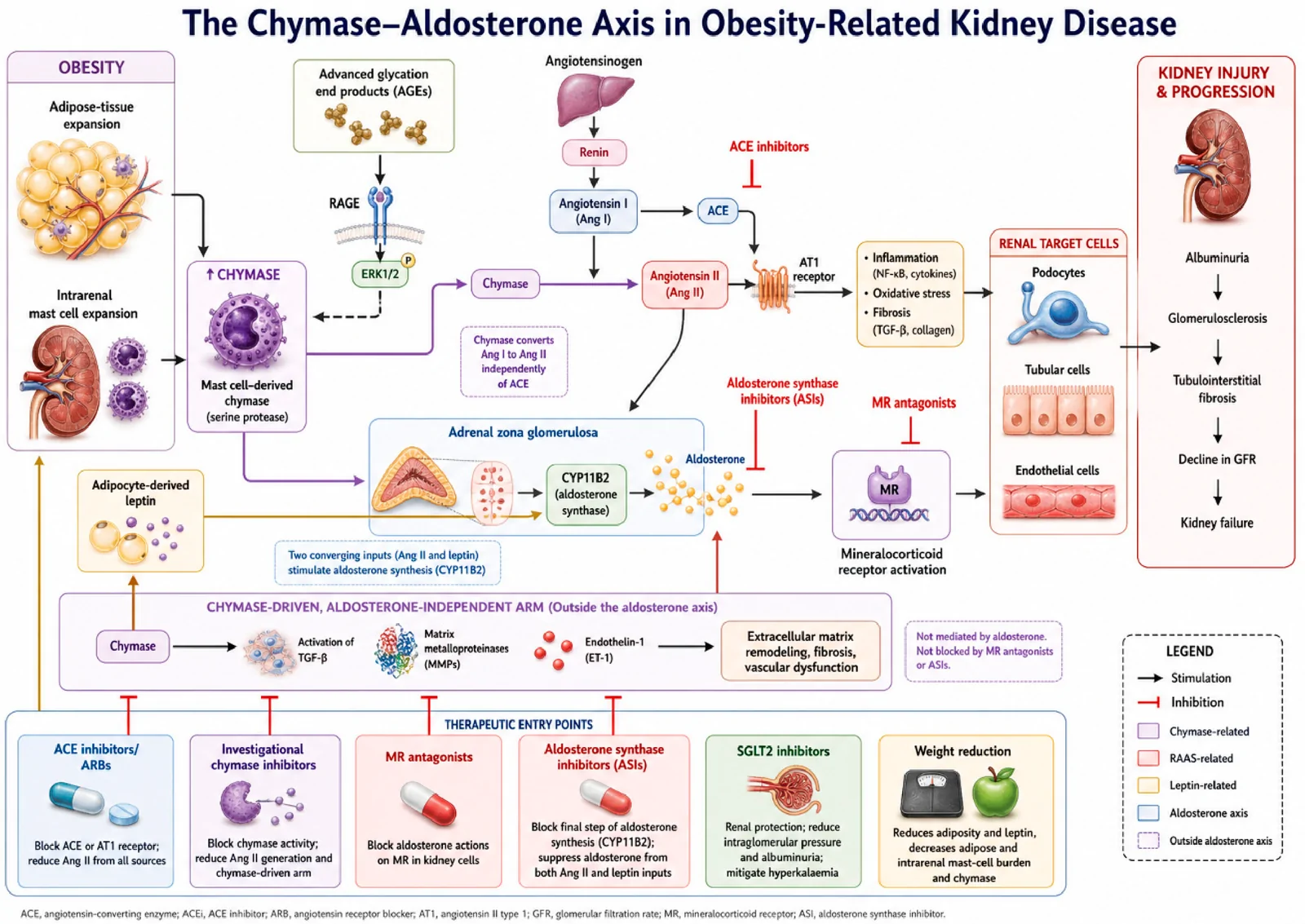
The chymase–aldosterone axis in obesity-related kidney disease. Schematic showing ACE-independent generation of angiotensin II by mast-cell chymase, the convergence of chymase-derived angiotensin II and adipocyte-derived leptin on adrenal aldosterone synthase (CYP11B2), and the resulting mineralocorticoid receptor-mediated injury to podocytes, tubular cells and endothelium. A chymase-driven, aldosterone-independent profibrotic arm is shown separately. Candidate therapeutic entry points are indicated. Solid arrows denote stimulation; blunted connectors denote pharmacological inhibition. ACE, angiotensin-converting enzyme; AGE, advanced glycation end product; ARB, angiotensin receptor blocker; ASI, aldosterone synthase inhibitor; AT1, angiotensin II type 1 receptor; CYP11B2, aldosterone synthase; ET-1, endothelin-1; MMP, matrix metalloproteinase; MR, mineralocorticoid receptor; MRA, mineralocorticoid-receptor antagonist; RAGE, receptor for AGE; SGLT2, sodium–glucose cotransporter-2; TGF-β, transforming growth factor-β.

A frequently overlooked determinant of aldosterone-pathway overactivity is chymase, a mast-cell serine protease that generates angiotensin II independently of ACE. Chymase is the most efficient and specific angiotensin I–to–angiotensin II–converting enzyme described in human tissue and is stored in mast-cell granules within the interstitium [84,85]. In the diabetic, hypertensive kidney, chymase is markedly upregulated in mesangial and vascular smooth-muscle cells and correlates with blood pressure and fibrosis far more strongly than ACE; advanced glycation end products further induce chymase through a RAGE–ERK1/2 pathway, linking the dysmetabolic milieu of obesity directly to alternative angiotensin II generation [21,86]. Critically, obese adipose tissue accumulates tryptase- and chymase-positive mast cells whose abundance tracks adipose fibrosis, macrophage infiltration and type 2 diabetes, so an individual with obesity carries an expanded, chymase-rich compartment in both fat and kidney [20] (Table 2, Figure 3).

#### 3.2.2. Intrarenal Chymase–Aldosterone Signalling in Podocytes and Tubules

Within the kidney itself, chymase is markedly upregulated in mesangial and vascular smooth-muscle cells in diabetic and hypertensive nephropathy, where its abundance correlates with blood pressure and with the degree of fibrosis considerably more strongly than that of ACE [86]. The angiotensin II generated locally acts through the AT1 receptor on podocytes, tubular epithelium and endothelium, and it also stimulates adrenal CYP11B2 expression. Chymase-derived angiotensin II and adipocyte-derived leptin therefore converge upon a single step—aldosterone synthesis—from two independent directions [18,19,86]. Aldosterone in turn activates the mineralocorticoid receptor, injuring podocytes through cytoskeletal disruption and oxidative stress, tubular cells through inflammatory and profibrotic signalling, and the endothelium through reduced nitric oxide bioavailability [56,82].

Because mast cells reside within the interstitium, this axis is anatomically a tubulointerstitial as much as a glomerular phenomenon, and it is considered again in that context in Section 4.2. Its specific relevance to obesity is that obese adipose tissue accumulates tryptase- and chymase-positive mast cells whose abundance tracks adipose fibrosis, macrophage infiltration, and T2DM, so that an individual with obesity carries an expanded, chymase-rich compartment in both fat and kidney [20].

#### 3.2.3. Therapeutic Implications of Chymase and Aldosterone Synthase Inhibition

The therapeutic consequences of this architecture are specific. Because aldosterone synthase inhibitors act at the final synthetic step, they suppress aldosterone generated downstream of both chymase-derived angiotensin II and leptin—an effect that ACE inhibition cannot achieve, since chymase bypasses ACE entirely. Mineralocorticoid receptor antagonists block the receptor but not either of the two upstream inputs. Neither class, however, addresses the aldosterone-independent arm of chymase activity, in which transforming growth factor-β, matrix metalloproteinases, and endothelin-1 are activated directly; that arm would require chymase inhibition itself, which remains investigational [21,86]. Weight reduction acts furthest upstream by lowering both circulating leptin and the adipose mast-cell burden, while SGLT2 inhibition provides additive albuminuria reduction and mitigates the hyperkalaemia risk of combined RAAS suppression [30,87]. The clinical and trial-level evidence for these classes is considered in Section 6.5.

### 3.3. Renal Lipotoxicity and Metabolic Stress

Renal lipid content reflects the balance of four processes—uptake, de novo synthesis, oxidation and export—each of which is disturbed in obesity. Delivery of non-esterified fatty acids to the kidney rises because visceral adipose tissue is lipolytically active and relatively resistant to the antilipolytic action of insulin, so that renal cells are exposed to chronic lipid oversupply. Proximal tubular epithelial cells and podocytes accumulate free fatty acids, triglycerides, ceramides and cholesterol esters when lipid uptake exceeds mitochondrial fatty-acid oxidation and safe storage capacity [61,88]. Uptake is facilitated by upregulation of the scavenger receptor CD36 and of fatty acid transport proteins and fatty acid-binding proteins on proximal tubular cells and podocytes, and by megalin–cubilin-mediated endocytosis of filtered albumin carrying bound fatty acids—a route that couples glomerular albumin leak directly to tubular lipid loading. Together with impaired peroxisome proliferator-activated receptor signalling, this favours intracellular lipid deposition and podocyte injury [61,89]. Saturated fatty acids, particularly palmitate, trigger endoplasmic-reticulum stress, mitochondrial dysfunction, reactive oxygen species generation and apoptosis [90].

The kidney also synthesises fatty acids locally, a contribution omitted from most accounts of renal lipotoxicity. Hyperinsulinaemia and substrate excess activate sterol regulatory element-binding protein-1c (SREBP-1c) and carbohydrate response element-binding protein (ChREBP), which induce acetyl-CoA carboxylase and fatty acid synthase and thereby drive de novo lipogenesis in tubular epithelial and glomerular cells. SREBP-2 concurrently promotes cholesterol synthesis and uptake, while suppression of the cholesterol efflux transporters ABCA1 and ABCG1 limits export. This synthetic arm helps to explain why lipid accumulates in the kidney even where dietary supply is not extreme, and why renal steatosis is more pronounced in T2DM, in which delivery and synthesis are increased simultaneously [61,88,91,92].

The proximal tubule is especially vulnerable because it relies heavily on oxidative metabolism. Suppression of peroxisome proliferator-activated receptor gamma coactivator-1alpha reduces mitochondrial biogenesis and fatty-acid oxidation, shifting tubular cells toward inefficient glycolysis and inflammatory signalling [93,94]. Lipid-laden tubular and interstitial cells recruit macrophages and amplify fibrosis through nuclear factor-kappaB, NLRP3 inflammasome and transforming growth factor-beta pathways [95,96]. Human biopsy evidence showing altered renal lipid metabolism and lipid-droplet accumulation supports the translational relevance of lipotoxicity [91,92].

### 3.4. Adipokines, Inflammation, and Oxidative Stress

#### 3.4.1. Adipokines and Metabolic Inflammation

Adipose tissue functions as a dysregulated endocrine and immune organ. Obesity is characterised by high leptin and relative leptin resistance, whereas adiponectin, which activates AMP-activated protein kinase and has anti-inflammatory and podocyte-stabilising effects, is usually reduced [97,98]. This imbalance strengthens pro-hypertensive and pro-fibrotic signalling and may capture renal risk beyond body mass index [65].

Visceral adipose tissue accumulates macrophages, T cells and senescent adipocytes that release tumour necrosis factor-alpha, interleukin-6, interleukin-1beta and monocyte chemoattractant protein-1 [99,100]. In the kidney, lipid stress, mitochondrial reactive oxygen species, uric acid and endotoxin converge on the NLRP3 inflammasome and nuclear factor-kappa B activation, promoting tubular injury and interstitial fibrosis [66,75]. Reactive oxygen species from NADPH oxidase, dysfunctional mitochondria and impaired antioxidant defences further reinforce senescence-associated secretory programmes [76].

#### 3.4.2. Oxidative Stress and Redox Imbalance

Oxidative stress deserves separate consideration because it is not a parallel mechanism but a convergence point at which the haemodynamic, lipotoxic, adipokine, gut-derived and mitochondrial arms of this network amplify one another. Reactive oxygen species in the obese kidney arise from several sources: NADPH oxidases, particularly NOX4 in podocytes and tubular epithelium; dysfunctional mitochondria, in which electron leak increases when fatty-acid oxidation is impaired; xanthine oxidase activity associated with hyperuricaemia; and uncoupled endothelial nitric oxide synthase, which generates superoxide in place of nitric oxide [66,75,76].

Antioxidant defence is simultaneously attenuated. Signalling through the Nrf2–Keap1 axis is blunted in obesity and diabetes, reducing expression of downstream antioxidant and detoxifying enzymes, while glutathione and superoxide dismutase capacity are depleted. The consequences are distributed across every renal compartment: podocyte cytoskeletal injury and detachment, tubular epithelial senescence, oxidative modification of lipids and proteins, reduced nitric oxide bioavailability with endothelial dysfunction, and activation of NLRP3 inflammasome and NF-κB signalling, which links redox imbalance directly to the inflammatory and fibrotic pathways described above [66,77,101].

A note of therapeutic realism is warranted. Direct antioxidant strategies have generally disappointed in kidney disease, whereas the agents with demonstrated renal benefit—SGLT2 inhibitors, GLP-1 receptor agonists and finerenone—reduce oxidative stress indirectly, by correcting the metabolic and neurohormonal drivers upstream of it rather than by scavenging reactive oxygen species. Redox imbalance is therefore best regarded as an amplifier to be addressed at its sources (Section 3.2, Section 3.3, Section 3.6 and Section 3.7) rather than as an independent therapeutic target [24,25,26].

### 3.5. Ghrelin, the Ghrelin–Leptin Axis, and Podocyte Protection

Ghrelin, the endogenous ligand of GHS-R1a and the principal stomach-derived orexigenic hormone, is a mechanistically important counterpart to leptin in obesity-related kidney disease [22,23]. It circulates in two forms: acyl ghrelin (AG), which requires serine-3 octanoylation by ghrelin O-acyltransferase for high-affinity binding to GHS-R1a and for the classical appetite and growth-hormone effects, and unacylated ghrelin (UAG, also termed des-acyl ghrelin), which predominates in plasma and remains metabolically active despite negligible GHS-R1a affinity and may counter-regulate the acylated form [102]. Unlike leptin, ghrelin is typically reduced in obesity and inversely associated with BMI, adiposity and insulin resistance, probably reflecting adaptation to chronic positive energy balance; hyperinsulinaemia and central ghrelin resistance further blunt ghrelin signalling [102,103,104].

The leptin–ghrelin axis is therefore doubly disturbed in obesity: leptin is elevated but resistant, whereas ghrelin is suppressed [22,105]. This shift may favour kidney injury because leptin promotes podocyte stress, inflammation and profibrotic signalling, while reduced ghrelin may weaken pathways supporting podocyte survival and glomerular filtration-barrier integrity [23]. Ghrelin also has renal antioxidant and anti-inflammatory actions; in angiotensin II–induced kidney injury, it preserved mitochondrial integrity and reduced ROS, senescence and fibrosis through a UCP2-dependent mechanism [106]. Thus, ghrelin suppression may remove an endogenous protective brake during obesity-related oxidative and inflammatory stress [107,108].

The opposing actions of leptin and of the two ghrelin forms are most clearly delineated across three effector domains (Table 3). In the glomerulus, leptin promotes podocyte stress, transforming growth factor-β signalling and profibrotic responses, whereas AG acting through GHS-R1a preserves mitochondrial integrity and reduces reactive oxygen species, senescence and fibrosis by a UCP2-dependent mechanism [82]; UAG does not bind GHS-R1a with high affinity and appears to act through a distinct, incompletely characterised receptor, so that its podocyte effects are reported but mechanistically less secure. In the tubule, leptin resistance is associated with impaired fatty-acid oxidation and lipid deposition in proximal tubular cells, whereas both ghrelin forms have been reported to improve insulin sensitivity and mitochondrial function, UAG showing the more consistent metabolic as opposed to orexigenic profile [102,104].

The third domain is autonomic, and it is here that the opposition is most direct. Leptin is sympathoexcitatory and contributes to obesity-related hypertension and renal sodium retention, whereas AG is sympathoinhibitory and vagally active [21,81]. The net renal effect of ghrelin nevertheless remains uncertain, because this sympathoinhibitory action is offset by a direct tubular one, as described below.

However, AG may also promote sodium retention. AG enhances distal-nephron sodium reabsorption, whereas renal ghrelin-receptor blockade induces natriuresis, suggesting potential haemodynamic harm in the obese kidney [109]. Clinical data remain limited and may be biphasic: ghrelin is low in uncomplicated obesity but may rise as CKD progresses because impaired renal clearance offsets obesity-related suppression [22]. Since most studies measure total rather than AG and UAG separately, and since the AG:UAG ratio rather than total ghrelin is the biologically meaningful variable, relevant changes may be obscured [102]. At present, ghrelin should be viewed as a plausible modulator and candidate biomarker, not an established therapeutic target [23].

### 3.6. Gut–Kidney Axis and Microbial Metabolites

The gut microbiome provides another interface between obesity and kidney injury. Obesity is associated with reduced microbial diversity, altered bile-acid metabolism, epithelial-barrier dysfunction and translocation of microbial products, while CKD intensifies dysbiosis through uraemia, intestinal oedema and reduced fibre intake [110,111]. Indoxyl sulphate and p-cresyl sulphate accumulate as kidney function declines and promote tubular oxidative stress, aryl hydrocarbon receptor activation, endothelial dysfunction and fibrosis [71,89]. Conversely, fibre-derived short-chain fatty acids may protect the kidney by improving barrier function, modulating immune tone and activating metabolic signalling [112].

These observations support a bidirectional model in which obesity and CKD reinforce each other through gut-derived inflammatory and metabolic signals [113]. Although probiotic, prebiotic, adsorbent and dietary strategies remain incompletely validated for hard renal outcomes, the gut-kidney axis is a modifiable translational frontier linked to fibre intake, obesity pharmacotherapy and uraemic-toxin biology [111,113].

### 3.7. Epigenetic Remodeling, Mitochondrial Dysfunction, and Senescence

Metabolic stress can create durable transcriptional memory in renal cells. DNA methylation, histone modification and non-coding RNAs regulate genes involved in podocyte structure, extracellular-matrix production, inflammation and epithelial repair [114,115]. miR-21 promotes maladaptive repair by targeting PTEN, SMAD7 and related anti-fibrotic pathways [116], whereas long non-coding RNAs such as NEAT1 remain plausible but less fully validated nodes in metabolic kidney injury [117].

Mitochondrial dysfunction links lipotoxicity, oxidative stress and sterile inflammation. Impaired fatty-acid oxidation, suppressed PGC-1alpha signalling and dysregulated fission-fusion balance reduce ATP generation and increase mitochondrial reactive oxygen species [94]. Damaged mitochondria release mitochondrial DNA, cardiolipin and other danger signals that activate NLRP3 and cGAS-STING pathways [77]. Repeated injury can drive renal cells into senescence, whose secretory phenotype sustains matrix deposition and immune recruitment [101,118].

Together, these pathways establish ORKD as a disorder in which haemodynamic stress, RAAS and mineralocorticoid signalling, lipid toxicity, inflammation, gut-derived toxins and mitochondrial-epigenetic memory interact over time. This integrated model supports early risk detection and combination strategies targeting both upstream adiposity and downstream kidney-injury pathways [54].

## 4. Renal Pathology of Obesity: Glomerular and Tubulointerstitial Phenotypes

### 4.1. Obesity-Related Glomerulopathy as a Clinicopathological Phenotype

Obesity-related glomerulopathy (ORG) is the prototypical biopsy-defined manifestation of obesity-associated renal injury and links excess adiposity to adaptive podocyte stress and progressive CKD [16,108]. Its defining lesion is glomerulomegaly, with or without perihilar focal segmental glomerulosclerosis (FSGS), reflecting structural adaptation to increased single-nephron filtration demand [108]. In some cases, mild mesangial expansion, nonspecific IgM or C3 trapping in sclerotic segments and variable tubulointerstitial fibrosis accompany the lesion, but the pattern remains most consistent with haemodynamic and metabolic stress rather than primary podocytopathy [16,119] (Table 4).

Accurate distinction from primary FSGS is essential because the entities share segmental sclerosis but differ in mechanism and treatment. Primary FSGS includes tip, cellular, collapsing, not-otherwise-specified and perihilar variants, whereas ORG is usually dominated by glomerulomegaly with perihilar accentuation [121]. Ultrastructurally, ORG typically shows partial, segmental foot-process effacement, in contrast to the diffuse effacement often seen in nephrotic primary FSGS [108,121]. Clinically, ORG usually presents with slowly progressive subnephrotic proteinuria, preserved serum albumin and absent or mild oedema, whereas primary FSGS more often causes nephrotic-range proteinuria and hypoalbuminaemia [108,121].

**Figure 4:**
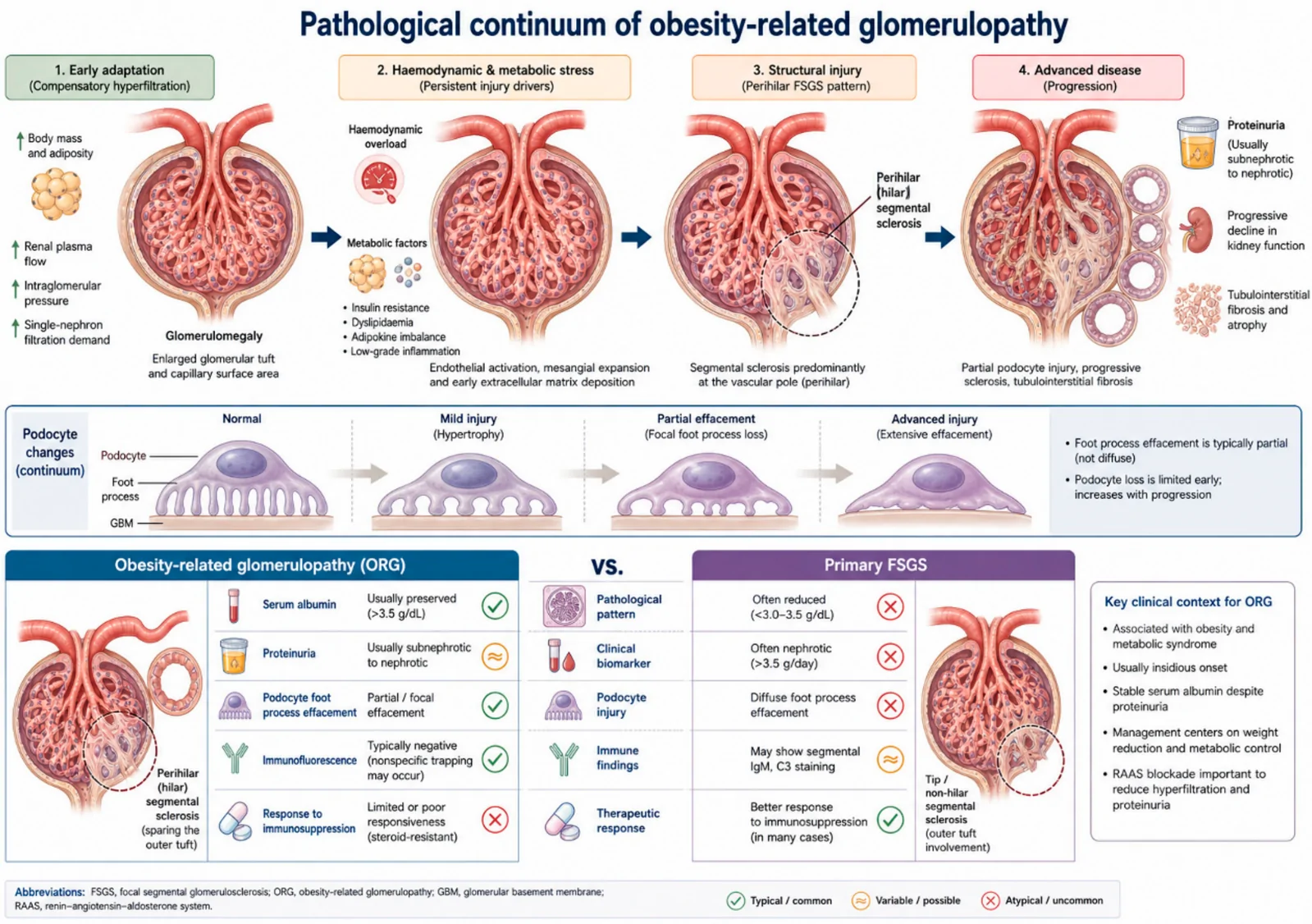
Pathological continuum of obesity-related glomerulopathy. Early obesity-related glomerular adaptation is characterized by glomerulomegaly and increased single-nephron filtration demand. Persistent hemodynamic and metabolic stress can produce perihilar FSGS, partial podocyte foot process effacement, proteinuria and progressive tubulointerstitial fibrosis. ORG is distinguished from primary FSGS by preserved serum albumin, partial rather than diffuse foot-process effacement and lower responsiveness to immunosuppression. FSGS, focal segmental glomerulosclerosis; ORG, obesity-related glomerulopathy.

The distinction has direct therapeutic consequences. ORG generally requires supportive and metabolic treatment rather than immunosuppression unless another glomerular disease is present [121,123]. Management should reduce intraglomerular pressure, proteinuria and adiposity through weight reduction, RAAS blockade and SGLT2 inhibition [127]. GLP-1 receptor agonists [25] and metabolic-bariatric surgery [128] provide additional options in selected patients. Long-term surveillance remains necessary because progression is favoured by higher baseline proteinuria, lower eGFR, tubulointerstitial fibrosis, persistent weight gain, hypertension and coexistent T2DM [16,124]. ORG should therefore be viewed not as an isolated glomerular lesion but as one phenotype within a broader continuum of adiposity-driven podocyte, tubular and interstitial injury (Figure 4) [4,84]. The tubulointerstitial component of that continuum, which determines functional decline at least as strongly as the glomerular lesion, is considered next. Obesity-related glomerulopathy and obesity-related tubulointerstitial injury should therefore be regarded not as separate entities but as two compartments of a single adiposity-driven process, coupled bidirectionally through hyperfiltration, filtered protein and lipid load, and shared neurohormonal and inflammatory mediators.

### 4.2. Obesity-Related Tubulointerstitial Injury

Attention to the glomerulus should not obscure the tubulointerstitium, in which the extent of fibrosis predicts functional decline at least as strongly as the glomerular lesion itself. Obesity injures this compartment through mechanisms that are partly shared with, and partly distinct from, those producing ORG, and they are best considered together as the second phenotype of ORKD.

The proximal tubule is the principal target, for metabolic reasons. It depends heavily on oxidative metabolism and possesses limited glycolytic reserve, so the lipid oversupply and impaired fatty-acid oxidation described in Section 3.3 translate directly into mitochondrial dysfunction, ATP depletion and lipid-droplet accumulation. Protein and lipid overload compounds this: megalin–cubilin-mediated endocytosis of filtered albumin and albumin-bound fatty acids imposes a lysosomal and endoplasmic-reticulum burden that activates NF-κB and transforming growth factor-β signalling, thereby transmitting glomerular albumin leak into tubular injury and coupling the two compartments mechanistically [61,88,129].

Sodium handling provides a second, tubule-centred route. Enhanced proximal reabsorption through sodium–glucose cotransporter-2 and the sodium–hydrogen exchanger NHE3 reduces distal sodium delivery to the macula densa, suppresses tubuloglomerular feedback and sustains afferent vasodilatation; in this sense the glomerular hyperfiltration of obesity is in part tubular in origin, which explains why SGLT2 inhibition is so well matched to the phenotype [13,55]. Compressive and hypoxic mechanisms act in parallel: perirenal and renal-sinus fat raise interstitial hydrostatic pressure and compress the vasa recta and lymphatics (Section 3.1.1), impairing medullary perfusion and producing the chronic hypoxia that drives hypoxia-inducible factor signalling and maladaptive repair.

Local neurohormonal and immune mechanisms complete the picture. Intrarenal RAAS activation and sympathetic overactivity are amplified in the interstitium by chymase-positive mast cells, which generate angiotensin II locally and independently of ACE (Section 3.2.2), while lipid-laden tubular cells recruit macrophages and activate NLRP3 inflammasome and NF-κB signalling [66,75,95,96]. Repeated injury drives epithelial cell-cycle arrest and senescence, whose secretory phenotype sustains myofibroblast recruitment and matrix deposition, so that interstitial fibrosis becomes the final common pathway of these processes [77,101,118]. Hyperuricaemia and, after malabsorptive bariatric procedures, enteric hyperoxaluria represent further tubulointerstitial consequences of the obese metabolic state [75,130].

Two translational implications follow. First, injury in this compartment may precede albuminuria, so tubular biomarkers—kidney injury molecule-1, neutrophil gelatinase-associated lipocalin and urinary epidermal growth factor—are candidates for earlier detection of ORKD than albuminuria alone permits, although none is yet validated for this purpose [50,131]. Second, several therapies with demonstrated renal benefit act at least partly on the tubule: SGLT2 inhibitors restore tubuloglomerular feedback and reduce tubular workload, GLP-1 receptor agonists attenuate tubular inflammation, and finerenone opposes mineralocorticoid receptor-driven interstitial fibrosis. The tubulointerstitial phenotype is therefore not merely a late consequence of glomerular disease but an independently modifiable target (Table 5).

## 5. Clinical Consequences Across CKD Care

### 5.1. Cardiovascular–Kidney–Metabolic Risk

Obesity and CKD are both major cardiovascular risk amplifiers [132]. Their co-occurrence increases the likelihood of atherosclerotic cardiovascular disease, heart failure, atrial fibrillation, resistant hypertension, and sudden cardiac death [132,133,134]. Mechanistically, this risk reflects overlapping RAAS activation, sympathetic drive, endothelial dysfunction, sodium retention, inflammation, and myocardial remodelling [135,136].

The term cardiovascular-kidney-metabolic (CKM) syndrome is useful because it emphasises shared pathobiology and supports integrated rather than siloed management [132,137,138]. In practice, the obesity-related CKD phenotype often requires simultaneous optimisation of blood pressure, albuminuria, weight, glycaemia, lipid status, sleep apnoea, heart failure risk, and kidney-protective pharmacotherapy [4,132,133].

### 5.2. Assessment and Pharmacological Complexity

Obesity complicates renal risk assessment [139,140]. Creatinine-based eGFR may be biased by muscle mass, while indexing GFR to 1.73 m^2^ can misrepresent absolute renal clearance in individuals with very large body surface area [139,140]. This bias is greatest in sarcopenic obesity, where low muscle mass causes creatinine-based eGFR to overestimate kidney function at the same time as BMI underestimates adiposity, so that risk is understated in both directions (Section 2.3). Cystatin C-based or combined creatinine–cystatin C equations may improve classification in selected patients, particularly when drug dosing, transplant assessment, or bariatric surgery planning depends on accurate renal function [51,54].

Drug dosing is also more complex because obesity alters volume of distribution, protein binding, hepatic metabolism, and renal tubular secretion, while CKD simultaneously reduces clearance and alters non-renal handling of many medications [141]. Aminoglycosides, vancomycin and selected antibiotics require particular caution because dosing is strongly influenced by body size, renal function, therapeutic drug monitoring requirements, and toxicity risk [142]. Anticoagulants also require caution in patients at extremes of weight or with renal impairment [143].

### 5.3. Dialysis and Transplantation

All kidney replacement therapy modalities are affected by obesity [52,144]. In haemodialysis, larger body size and altered body composition can complicate Kt/V-based urea-clearance assessment and prescription, while vascular access creation, maturation and cannulation may be technically challenging [145,146]. In peritoneal dialysis, abdominal adiposity and dialysate-related increases in intra-abdominal pressure may raise the risk of dialysate leaks, hernias, catheter-related mechanical complications, and technique failure [42,147]. In kidney transplantation, obesity is associated with delayed graft function, wound complications, post-transplant diabetes, and longer waitlisting; many centres also use BMI thresholds as relative or absolute barriers to transplantation [148,149]. These practices require careful ethical consideration because BMI thresholds can reduce access to transplantation without fully capturing surgical risk, body composition or the likelihood of successful weight intervention [150,151].

## 6. Therapeutic Strategies

**Figure 5:**
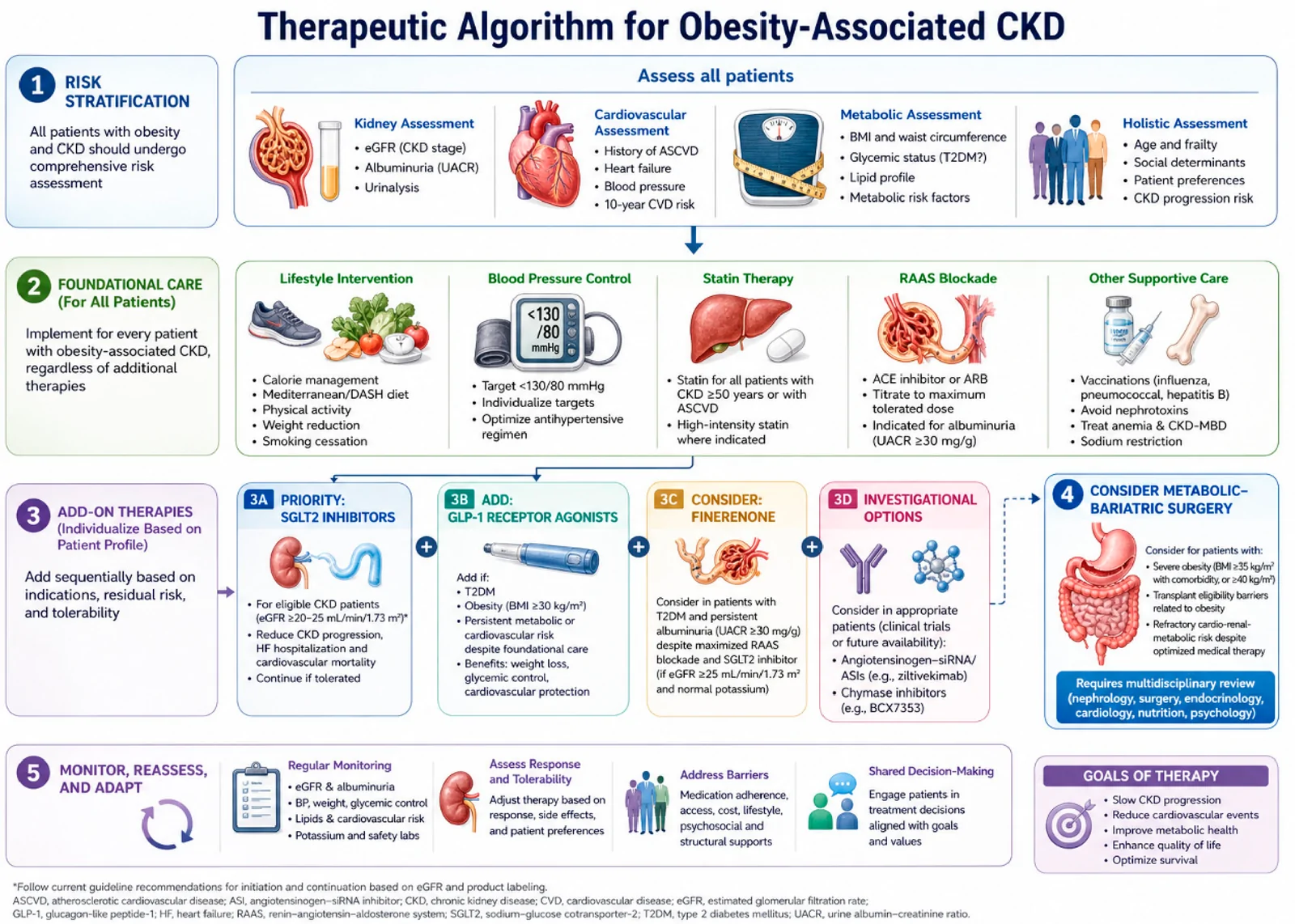
Therapeutic algorithm for ORKD. All patients should receive CKD risk stratification with eGFR, albuminuria and cardiovascular assessment. Foundational care includes lifestyle intervention, blood pressure control, statin therapy where indicated and RAAS blockade for albuminuria. SGLT2 inhibitors are prioritised for eligible CKD patients; GLP-1 receptor agonists are added for T2DM, obesity or persistent metabolic risk; finerenone is considered for residual albuminuria in T2DM; and metabolic-bariatric surgery is considered for severe obesity, transplant eligibility barriers or refractory cardio-renal-metabolic risk after multidisciplinary review.

Correct diagnosis sets the stage for the increasingly potent and mechanism-matched therapies summarised in Table 6 and Table 7 and Figure 5, which map the major classes onto their evidence base, renal signal and relevance to adiposity-driven cardiorenal-metabolic risk. Mechanism-based therapy for ORKD should be layered rather than single-pathway, because excess adiposity combines glomerular hyperfiltration, adipose-derived RAAS activation, metabolic inflammation, tubular stress and cardiovascular-kidney-metabolic risk [4,54]. Treatment selection should integrate eGFR, albuminuria, diabetes and heart-failure status, obesity severity, nutritional reserve, transplant candidacy and patient preference [4,54].

Two principles should govern how the mechanisms of Section 3 and Section 4 are translated into treatment. The first is that therapy must be layered rather than sequential. ORKD is a multi-compartment disorder in which haemodynamic, metabolic, neurohormonal and anatomical drivers operate concurrently, so that any single-pathway intervention leaves the remaining drivers intact; this is the pharmacological rationale for combining agents whose mechanisms differ rather than escalating within one class. The second is that the strength of evidence differs sharply between classes, and this difference should be visible in clinical reasoning: some interventions are supported by dedicated kidney-outcome trials, others by secondary or post hoc renal analyses, and others by mechanistic plausibility alone. Table 6 maps each mechanism onto the class that targets it, the level of human evidence that the mechanism is actually modified, and the residual gap.

### 6.1. Lifestyle, Diet, and Exercise

#### 6.1.1. Weight Reduction and Physical Activity

Lifestyle intervention remains the foundation of care, but it should be realistic and guided by kidney-nutrition expertise. A 5–10% weight reduction can improve blood pressure, albuminuria, insulin resistance and inflammatory biomarkers, whereas durable effects on hard kidney outcomes remain less certain [54,163]. Exercise, especially combined aerobic and resistance training, can reduce visceral adiposity and improve functional capacity, but effects on proteinuria and blood pressure are heterogeneous [164,169]. Dietary prescriptions should balance caloric restriction, sodium reduction, fibre intake and avoidance of ultra-processed foods against the risks of protein-energy wasting and sarcopenia, particularly in older adults and advanced CKD [49,54].

#### 6.1.2. Dietary Patterns and Kidney Protection

Dietary composition deserves attention in its own right, since diet is the most widely applicable of the interventions considered here and acts on several of the mechanisms described in Section 3 simultaneously. Evidence is strongest for dietary patterns rather than single nutrients. Mediterranean, DASH and plant-dominant patterns are each associated with slower eGFR decline and lower incident CKD in cohort studies, and they share a common profile: high fibre, a high ratio of unsaturated to saturated fat, low sodium, limited refined carbohydrate and moderate animal protein [49,54,163].

The mechanistic correspondence with Section 3 is direct. Reducing saturated fat limits the substrate available for renal lipotoxicity and for de novo lipogenesis (Section 3.3). Fermentable fibre increases short-chain fatty acid production and lowers generation of indoxyl sulphate and p-cresyl sulphate, engaging the gut–kidney axis (Section 3.6). Sodium restriction lowers intraglomerular pressure and improves the antiproteinuric response to RAAS blockade. A lower dietary acid load reduces tubulointerstitial injury (Section 4.2). Higher consumption of ultra-processed foods is associated with incident CKD independently of BMI, implicating additives, advanced glycation end products and sodium load in addition to energy density—a link of particular interest here, since advanced glycation end products induce chymase through RAGE–ERK1/2 signalling (Section 3.2.1) [21].

Protein intake requires a balanced judgement. High-protein diets are effective for weight loss and for preserving lean mass, but they increase glomerular hyperfiltration and may be inadvisable in individuals already hyperfiltering; conversely, protein restriction in advanced CKD risks protein-energy wasting and sarcopenia, particularly in older adults (Section 2.3). Plant-dominant approaches, in which moderate protein is obtained largely from plant sources, offer a possible reconciliation and additionally lower dietary acid and phosphate load [49,54].

The strength of this evidence should not be overstated. Most dietary data in CKD are observational and susceptible to confounding by socioeconomic position and by overall health behaviour; randomised dietary trials with hard renal endpoints remain scarce; and adherence attenuates over time. Dietary recommendations in this population therefore rest on a weaker evidence base than the pharmacological recommendations that follow. Diet nonetheless retains a distinctive role, because it is universally accessible, addresses several mechanisms at once, and remains the foundation upon which incretin-based and surgical weight-loss strategies are built.

### 6.2. SGLT2 Inhibitors

SGLT2 inhibitors are well matched to the hyperfiltration phenotype of obesity-related CKD because they reduce proximal sodium-glucose reabsorption, restore tubuloglomerular feedback and lower intraglomerular pressure [55]. DAPA-CKD [127] and EMPA-KIDNEY [24] showed major reductions in CKD progression across diabetic and selected non-diabetic CKD populations, supporting this class as foundational cardiorenal therapy. Practical use should follow product labelling and CKD guidelines, with attention to eGFR thresholds, early eGFR dip, volume status, genital mycotic infection, perioperative withholding and rare ketoacidosis risk [54,170].

### 6.3. GLP-1 Receptor Agonists and Dual Incretin Agonism

GLP-1 receptor agonists complement SGLT2 inhibition by targeting adiposity, appetite regulation, glycaemia, inflammation and albuminuria. FLOW demonstrated that semaglutide reduced a composite kidney and cardiovascular death endpoint in type 2 diabetes with CKD, while obesity trials support clinically meaningful weight loss [25,155]. Dual GIP/GLP-1 receptor co-agonism extends this approach. Tirzepatide produces the most pronounced weight reduction of any currently approved pharmacological agent, and analyses from its cardiometabolic and obesity programmes have reported favourable effects on albuminuria and on eGFR slope, including in participants with reduced kidney function [127,157]. These renal endpoints were, however, secondary or post hoc rather than primary, and no dedicated kidney-outcome trial of a GIP/GLP-1 co-agonist has yet been reported. The evidence for this class should therefore be regarded as hypothesis-generating, and it is distinct in kind from the dedicated kidney-outcome evidence available for semaglutide. Whether the renal benefit of incretin-based therapy is mediated principally by weight loss, by glycaemic improvement or by weight-independent anti-inflammatory and haemodynamic actions remains unresolved and is a priority for mechanistic study.

### 6.4. RAAS Blockade and Mineralocorticoid Receptor Antagonism

ACE inhibitors and angiotensin receptor blockers remain essential in proteinuric CKD because they reduce efferent arteriolar tone, intraglomerular pressure and albuminuria [59,159,160]. This rationale is strengthened in ORKD by adipose-derived RAAS activation and intraglomerular hypertension [125,161]. Doses should be titrated to the maximally tolerated approved range while monitoring blood pressure, serum creatinine and potassium, and dual RAAS blockade should generally be avoided [171].

Finerenone extends RAAS-directed care by antagonising mineralocorticoid-receptor-driven inflammation and fibrosis. In FIDELIO-DKD [26] and FIGARO-DKD [27], finerenone reduced kidney and cardiovascular outcomes in type 2 diabetes with albuminuric CKD despite background RAAS blockade. Pooled analysis of the two trials confirmed consistent kidney and cardiovascular benefit across the albuminuria and eGFR spectrum, and combination with SGLT2 inhibition has since been examined with the aim of achieving additive albuminuria reduction while limiting hyperkalaemia. Two limitations should be stated plainly. First, the pivotal evidence base for finerenone is confined to type 2 diabetes, so its application to non-diabetic ORKD is an extrapolation rather than a demonstrated indication—a gap noted again in Section 7 and Section 8. Second, hyperkalaemia surveillance remains necessary, particularly in advanced CKD or combination therapy [57].

### 6.5. Aldosterone Synthase Inhibition and the Chymase Pathway

ASIs inhibit CYP11B2 to lower aldosterone biosynthesis upstream of the mineralocorticoid receptor; the central pharmacological challenge is achieving selectivity over CYP11B1, which shares roughly 93% sequence identity and is required for cortisol synthesis. In the phase 2 BrigHTN trial, the selective ASI baxdrostat produced dose-dependent reductions in plasma aldosterone and clinically meaningful blood-pressure lowering in treatment-resistant hypertension without affecting cortisol [28]. Evidence specific to the kidney is accruing: in 586 adults with albuminuric CKD on maximally tolerated RAAS inhibition, the highly selective ASI vicadrostat (BI 690517) reduced the urine albumin-to-creatinine ratio by approximately 40% at the 10-mg dose, with additive benefit when combined with empagliflozin and with hyperkalaemia as the principal safety signal, attenuated by SGLT2 inhibition [87]. These findings underpin the large EASi-KIDNEY outcomes trial of vicadrostat plus empagliflozin across diabetic and non-diabetic CKD [30]. For the obesity phenotype specifically, ASIs are attractive because they suppress aldosterone arising from both angiotensin II–dependent and adipocyte/leptin–dependent inputs [19,83].

Two features make the chymase–aldosterone relationship central to therapeutic reasoning. First, chymase acts upstream of aldosterone: the angiotensin II it generates is itself a stimulus for adrenal CYP11B2 expression and aldosterone synthesis, so in obesity chymase-derived angiotensin II and leptin converge on aldosterone production. Because ASIs act at the final synthetic step, they blunt aldosterone generated downstream of chymase-derived angiotensin II—an effect ACE inhibitors cannot achieve—helping to explain residual aldosterone-mediated injury (“aldosterone escape”) during conventional RAAS blockade and the additional albuminuria reduction observed when synthesis is suppressed directly [86,87]. Second, chymase also injures the kidney independently of aldosterone, generating AT1-receptor–mediated inflammation and fibrosis and activating transforming growth factor-β, matrix metalloproteinases and endothelin-1; these actions lie outside the aldosterone axis, so neither a mineralocorticoid-receptor antagonist nor an ASI fully neutralises chymase-driven damage [21,86].

These considerations favour a combinatorial approach in ORKD: layering aldosterone synthase inhibition onto background RAAS and SGLT2 inhibition simultaneously addresses aldosterone arising from angiotensin II–dependent, chymase-dependent, and leptin-dependent sources, while SGLT2 inhibition mitigates the attendant hyperkalaemia risk [30,87]. Direct chymase inhibition remains investigational but is a rational future target for the aldosterone-independent component described in Section 3.2.3. Its slow clinical translation reflects two specific obstacles: marked species differences in chymase biology, such that rodent chymases differ in substrate specificity from human chymase and standard models therefore predict human responses poorly; and the difficulty of achieving selectivity within the wider chymase family and against related serine proteases. Weight reduction itself lowers leptin and adipose mast-cell burden, and weight reduction itself lowers leptin and adipose mast-cell burden, attenuating both arms of this system [20]. Dedicated studies of aldosterone synthase—and chymase—inhibition in obesity-defined kidney cohorts are now needed to translate this rationale into practice.

### 6.6. Metabolic–Bariatric Surgery

Metabolic-bariatric surgery produces the most durable weight loss for severe obesity and can improve albuminuria, blood pressure, glycaemia and measured or estimated GFR in early-to-moderate CKD [172,173]. It is particularly relevant when obesity limits transplant candidacy or impedes CKD, diabetes or cardiovascular-risk control [167,173]. Sleeve gastrectomy is often preferred for transplant candidates because it avoids intestinal bypass, whereas Roux-en-Y gastric bypass can cause enteric hyperoxaluria and, rarely, oxalate nephropathy [130]. Advanced CKD requires multidisciplinary assessment because perioperative acute kidney injury, malnutrition, fluid shifts and medication changes can offset benefit [168].

## 7. Research Priorities for Precision Nephrology

The next phase of obesity-related CKD research should move beyond BMI toward mechanism-guided precision nephrology (Table 8). First, adipose-depot biology requires better definition: perirenal, renal sinus, visceral and subcutaneous fat are unlikely to carry equivalent renal risk, and studies combining standardised imaging with adipose transcriptomics and longitudinal renal outcomes are needed to identify injurious depots [17,174]. Second, phenotype-specific trials should determine which patients benefit most from SGLT2 inhibitors, GLP-1 receptor agonists, finerenone or metabolic-bariatric surgery, stratified by obesity phenotype, albuminuria, diabetes status and body composition [4,25]. Third, clinically actionable biomarkers are needed to distinguish lipotoxic, inflammatory and fibrotic injury; urine or plasma proteomics, metabolomics and spatial single-cell validation could convert mechanistic pathways into treatment-selection tools [50,131]. Fourth, sarcopenic obesity requires trials that combine safe weight loss with resistance exercise, nutrition support and body-composition endpoints to avoid worsening frailty or muscle loss [49,165]. Fifth, ageing with obesity and T2DM should be studied as a distinct high-risk phenotype rather than as three separate exposures, using cystatin C-based filtration estimates, body-composition measurement and functional endpoints, since older adults with frailty or sarcopenia are systematically underrepresented in existing trials. Sixth, perirenal and renal-sinus fat thickness should be evaluated prospectively as a stratifying variable, given that the mechanical and paracrine actions of these depots (Section 3.1.1) predict responses to different interventions. Finally, transplant access should be re-evaluated through prospective centre-level studies that integrate surgical risk, functional status and body composition rather than rigid BMI cut-offs [175,176]. Together, these priorities would align depot-specific biology, validated biomarkers and phenotype-matched therapy with patient-centred outcomes [4].

## 8. Limitations of the Evidence Base

Several limitations temper the current interpretation. Much of the mechanistic literature is derived from high-fat-diet or genetic obesity models that do not fully reproduce the human phenotype, which typically includes ageing, hypertension, T2DM, sleep apnoea, dyslipidaemia, medications and social determinants of health [181,182]. Human biopsy data are informative but inherently subject to selection bias because kidney biopsy is not routinely performed in uncomplicated obesity or mild albuminuria [13,16]. Large trials of SGLT2 inhibitors, GLP-1 RAs and finerenone were not designed specifically around ORG or perirenal adiposity, so extrapolation to non-diabetic obesity-related CKD requires caution [24,25]. In addition, there is as yet no consensus terminology or diagnostic threshold for fatty kidney comparable to that now established for steatotic liver disease, and most supporting human data are cross-sectional, so the direction of the association between renal lipid content and functional decline is not established. The evidence supporting dietary intervention rests largely on observational cohorts susceptible to confounding by socioeconomic position and overall health behaviour, and randomised dietary trials with hard renal endpoints remain scarce. Older adults with frailty, sarcopenia or reduced eGFR are frequently excluded from the trials on which current recommendations rest, so the evidence is weakest precisely where absolute risk is highest. Finally, BMI remains the most commonly reported exposure, despite its inability to distinguish visceral fat, ectopic fat, muscle mass and fluid overload [48,52].

These limitations do not weaken the central conclusion that obesity is a causal and modifiable contributor to CKD; rather, they highlight the need for better phenotyping [8,183]. The next phase of the field should connect clinical outcomes with molecular mechanisms in human tissues and should test whether mechanism-guided therapies can prevent the transition from adaptive hyperfiltration to irreversible fibrosis [16,184].

## 9. Conclusions

In summary, obesity is an independent and modifiable driver of the incidence, progression and clinical complexity of chronic kidney disease, acting through a self-reinforcing, multi-compartment network rather than any single lesion [4,9]. Glomerular hyperfiltration, adipose-derived RAAS activation, lipotoxicity, adipokine imbalance, inflammation, oxidative stress, gut dysbiosis, epigenetic remodelling, mitochondrial dysfunction and senescence converge on podocyte stress, tubular failure and interstitial fibrosis, spanning a continuum from early albuminuria to obesity-related glomerulopathy and progressive CKD. Two non-canonical routes deserve emphasis: leptin directly upregulates adrenal aldosterone synthase (CYP11B2), and mast-cell chymase generates angiotensin II independently of angiotensin-converting enzyme, amplifying injury that conventional RAAS blockade incompletely addresses. Counter-regulating this, obesity-related suppression of ghrelin may withdraw an antioxidant, anti-inflammatory and podocyte-protective signal, making it a plausible modulator and candidate biomarker warranting prospective study. ORG and obesity-related tubulointerstitial injury should be regarded not as separate entities but as two compartments of a single adiposity-driven process, coupled bidirectionally through hyperfiltration, filtered protein and lipid load, and shared neurohormonal and inflammatory mediators. Mechanism-matched therapies—SGLT2 inhibitors, GLP-1 receptor agonists, finerenone, lifestyle intervention, metabolic-bariatric surgery and aldosterone synthase inhibitors—now enable a precision cardiovascular-kidney-metabolic framework matching adipose-depot biology and biomarkers to phenotype-specific treatment.

## Figures and Tables

**Table 1:** Molecular and physiological pathways link obesity to CKD. This table summarises the major haemodynamic, endocrine, metabolic, inflammatory, gut-derived, epigenetic and mitochondrial pathways through which obesity promotes glomerular, tubular, endothelial and interstitial injury in CKD.

Pathway	Principal Mediators	Renal Target	Translational Implication	References
Haemodynamic stress	Hyperfiltration, renal sinus/perirenal fat, sympathetic activation, tubular sodium retention	Podocytes, glomerular capillaries	Supports early albuminuria screening and therapies that reduce intraglomerular pressure	Pathway/mediators/renal target: [7,13]. Translational implication: [54,55,56,57].
Adipose RAAS and aldosterone	Angiotensin II, AT1 receptor signalling, aldosterone, mineralocorticoid receptor activation	Glomerulus, tubules, endothelium, interstitium	Rationale for ACE inhibitor/ARB therapy and finerenone in albuminuric disease	Pathway/mediators/renal target: [7,58]. Translational implication: [26,57,59].
Renal lipotoxicity	Free fatty acids, CD36, FABPs, ceramides, ER stress, and impaired FAO	Proximal tubules and podocytes	Links weight loss and metabolic therapy to reduced tubular stress and fibrosis	Pathway/mediators/renal target: [60,61,62] Translational implication: [25].
Adipokine imbalance	High leptin, low adiponectin, resistin, chemerin, lipocalin-2	Podocytes, mesangium, vasculature	May identify biomarkers or targets beyond BMI	Pathway/mediators/renal target: [63,64,65]. Translational implication/biomarkers: [50].
Inflammation and oxidative stress	M1 macrophages, TNF-α, IL-6, NLRP3, NF-κB, NOX4, ROS	Endothelium, tubules, interstitium	Explains persistent CKD risk despite glycaemic and blood pressure control	Pathway/mediators/renal target: [66,67,68]. Translational implication: [25,26].
Gut-kidney axis	Indoxyl sulphate, p-cresyl sulphate, LPS/TLR4, reduced SCFAs	Tubules, immune cells, endothelium	Supports microbiome, fibre and uraemic toxin-directed strategies	Pathway/mediators/renal target: [69,70]. Translational implication: [71].
Epigenetic and mitochondrial injury	DNA methylation, miR-21, lncRNAs, PGC-1α suppression, mtDNA release	Tubules, fibroblasts, immune cells	Basis for precision biomarkers and anti-fibrotic target discovery	Pathway/mediators/renal target: [72,73,74]. Translational implication/biomarkers: [50].
Oxidative stress and redox imbalance	NOX4, mitochondrial ROS, xanthine oxidase, uncoupled eNOS, attenuated Nrf2–Keap1 defence	Podocytes, tubular epithelium, endothelium	Convergence point amplifying the other pathways; addressed indirectly by SGLT2 inhibitors, GLP-1 RAs and finerenone rather than by direct antioxidants	See Section 3.4.2 [66,75,76,77]

Abbreviations: RAAS, renin-angiotensin-aldosterone system; AT1, angiotensin II type 1; FABP, fatty acid-binding protein; ER, endoplasmic reticulum; FAO, fatty-acid oxidation; TNF, tumour necrosis factor; IL, interleukin; NF-κB, nuclear factor-κB; NOX4, NADPH oxidase 4; ROS, reactive oxygen species; LPS, lipopolysaccharide; SCFA, short-chain fatty acid; lncRNA, long non-coding RNA; PGC-1α, peroxisome proliferator-activated receptor γ coactivator-1α; mtDNA, mitochondrial DNA.

**Table 2:** The chymase system in obesity-related kidney disease. This table summarises the components of the mast-cell chymase pathway and their relevance to ORKD, including chymase as an ACE-independent source of angiotensin II, its induction in adipose tissue and the diabetic/hypertensive kidney, its convergence on aldosterone synthesis, its aldosterone-independent profibrotic actions, and the corresponding therapeutic implications.

Component/Mechanism	Molecular Action	Evidence in Obesity/Kidney	Therapeutic Implication	Ref.
Mast-cell chymase (CMA1)	ACE-independent conversion of Ang I to Ang II; most efficient, specific Ang II-forming serine protease; stored in mast-cell granules	Identified in human heart; localises to interstitial mast cells	Generates Ang II not blocked by ACE inhibitors; rationale for chymase inhibition	[84,85]
Adipose mast-cell expansion	Accumulation of tryptase+/chymase+ mast cells and CMA1 in visceral fat	Correlates with adipose fibrosis, macrophage infiltration and T2D	Weight reduction lowers mast-cell burden and substrate	[20]
AGE–RAGE–ERK1/2 induction	AGEs induce chymase expression and chymase-dependent Ang II generation	Shown in diabetic vasculature/vascular smooth-muscle cells	Links dysmetabolic milieu of obesity to local Ang II	[21]
Renal chymase up-regulation	~10–15-fold increase in mesangial and vascular smooth-muscle cells with matrix deposition	Human diabetic/hypertensive nephropathy; correlates with BP and fibrosis	Explains incomplete protection by ACE inhibition	[86]
Chymase → Ang II → CYP11B2 arm	Chymase-derived Ang II stimulates adrenal aldosterone synthase	Converges with leptin-driven CYP11B2 in obesity	Captured at the synthetic step by ASIs	[19,83,86,87]
Aldosterone-independent injury	Chymase activates TGF-β, MMPs and big-endothelin → ET-1; AT1-mediated inflammation/fibrosis	Profibrotic actions outside the aldosterone axis	Not neutralised by MRA or ASI; needs chymase inhibition	[21,86]
Therapeutic integration	Combine ASI with RAAS and SGLT2 inhibition; chymase inhibition investigational	Vicadrostat reduced UACR ~40%, additive with empagliflozin	Multi-arm RAAS suppression; SGLT2i mitigates hyperkalaemia	[30,87]

Abbreviations: ACE, angiotensin-converting enzyme; Ang II, angiotensin II; AT1, angiotensin II type 1 receptor; AGE, advanced glycation end product; RAGE, receptor for AGE; ERK1/2, extracellular signal-regulated kinase 1/2; CYP11B2, aldosterone synthase; MR, mineralocorticoid receptor; MRA, mineralocorticoid-receptor antagonist; ASI, aldosterone synthase inhibitor; SGLT2, sodium-glucose cotransporter-2; TGF-β, transforming growth factor-β; MMP, matrix metalloproteinase; ET-1, endothelin-1; UACR, urine albumin-to-creatinine ratio; T2D, type 2 diabetes; →, progress to.

**Table 3:** Opposing actions of leptin, acyl ghrelin and unacylated ghrelin on the kidney in obesity. This table contrasts the three mediators across the effector domains most relevant to obesity-related kidney disease.

Domain	Leptin (Elevated in Obesity)	Acyl Ghrelin, AG (Suppressed in Obesity)	Unacylated Ghrelin, UAG
Receptor and signaling	Leptin receptor (ObR); JAK–STAT3 and PI3K signalling	GHS-R1a; UCP2-dependent antioxidant signalling	Negligible GHS-R1a affinity; distinct, uncharacterised receptor
Podocyte injury	Promotes podocyte stress, TGF-β signalling and fibrosis	Preserves mitochondrial integrity; reduces ROS, senescence and fibrosis	Protective effects reported; mechanisms are less secure
Tubular lipid accumulation	Leptin resistance associated with impaired FAO and lipid deposition	Improves mitochondrial function and insulin sensitivity	Consistent metabolic rather than orexigenic profile
Sympathetic activation	Sympathy-excitatory; hypertension and sodium retention	Sympathoinhibitory; vagally active	Minimal direct autonomic effect
Renal sodium handling	Enhances tubular sodium reabsorption	Enhances distal-nephron sodium reabsorption (potentially harmful)	Not established
Behaviour in obesity and CKD	Elevated, with receptor resistance	Suppressed in obesity; may rise as CKD advances owing to reduced clearance	Predominant circulating form; rarely measured separately

Abbreviations: AG, acyl ghrelin; CKD, chronic kidney disease; FAO, fatty-acid oxidation; GHS-R1a, growth hormone secretagogue receptor 1a; ObR, leptin receptor; ROS, reactive oxygen species; TGF-β, transforming growth factor-β; UAG, unacylated ghrelin; UCP2, uncoupling protein 2.

**Table 4:** Clinicopathological comparison of ORG and primary FSGS. This table compares the dominant pathological, ultrastructural and clinical features of obesity-related glomerulopathy and primary FSGS to support accurate diagnosis and treatment selection.

Feature	Obesity-Related Glomerulopathy	Primary FSGS	Clinical Consequence	References
Dominant lesion	Glomerulomegaly with or without perihilar FSGS	Tip, cellular, collapsing, NOS or perihilar variants	ORG suggests adaptive/metabolic injury rather than primary podocytopathy	ORG [15,108,119]. Primary FSGS [120,121,122]. Clinical consequence: [121].
Foot process effacement	Usually partial and segmental	Often diffuse in nephrotic primary FSGS	Helps avoid inappropriate immunosuppression	ORG [15,108]. Primary FSGS [121,123]. Clinical consequence: [121].
Proteinuria/albumin	Often subnephrotic; serum albumin preserved	Frequently nephrotic; hypoalbuminaemia common	Supports supportive and metabolic therapy first	ORG [15,108,119]. Primary FSGS [121,123]. Clinical consequence: [121].
Natural history	Slow-to-moderate progression; accelerated by T2DM, hypertension and fibrosis	Variable; collapsing variant often aggressive	Requires long-term albuminuria, eGFR and weight monitoring	ORG [15,108,124]. Primary FSGS [120,121,122]. Clinical consequence: [121].
Treatment emphasis	Weight loss, RAAS blockade, SGLT2 inhibitor, GLP-1 RA; surgery in selected cases	Immunosuppression plus supportive care according to subtype	Correct diagnosis changes management strategy	ORG management: [25,125,126,127]. Primary FSGS management: [121,123].

Abbreviations: ORG, obesity-related glomerulopathy; FSGS, focal segmental glomerulosclerosis; NOS, not otherwise specified; T2DM, type 2 diabetes mellitus; RAAS, renin-angiotensin-aldosterone system; SGLT2, sodium-glucose cotransporter-2; GLP-1 RA, glucagon-like peptide-1 receptor agonist.

**Table 5:** The two compartments of obesity-related kidney disease: glomerular and tubulointerstitial phenotypes.

Feature	Glomerular Phenotype (ORG, Section 4.1)	Tubulointerstitial Phenotype (Section 4.2)
Dominant lesion	Glomerulomegaly with or without perihilar FSGS	Proximal tubular lipid accumulation, atrophy and interstitial fibrosis
Principal mediators	Hyperfiltration, podocyte stress, leptin, aldosterone, angiotensin II	Lipotoxicity and de novo lipogenesis, filtered protein load, hypoxia, chymase-derived angiotensin II, senescence
Coupling to the other compartment	Albumin leak delivers protein and lipid to the tubule	Proximal sodium reabsorption suppresses tubuloglomerular feedback and sustains hyperfiltration
Clinical markers	Albuminuria; subnephrotic proteinuria with preserved serum albumin	Candidate tubular markers (KIM-1, NGAL, uEGF); eGFR decline; fibrosis on biopsy
Prognostic weight	Predicts progression, but less strongly than fibrosis	Interstitial fibrosis is the strongest histological predictor of functional decline
Therapeutic emphasis	Weight reduction, RAAS blockade, SGLT2 inhibition, GLP-1 RAs	SGLT2 inhibition, finerenone, correction of hypoxia and lipid load; chymase-directed strategies investigational

**Table 6:** Mapping mechanisms of obesity-related kidney disease onto therapeutic classes. This table links each mechanism described in Section 3 to the therapeutic class that targets it, the strength of human evidence that the mechanism is modified, and the principal outstanding gap.

Mechanism (Section)	Therapeutic Class	Strength of Human Evidence	Residual Gap
Glomerular hyperfiltration and tubuloglomerular feedback (Section 3.1 and Section 4.2)	SGLT2 inhibitors	Dedicated kidney-outcome RCTs in diabetic and non-diabetic CKD	Few participants enrolled by obesity phenotype; ORG-specific data lacking
Mechanical compression by perirenal and renal-sinus fat (Section 3.1.1)	Weight reduction; metabolic-bariatric surgery	Observational; imaging shows depot reduction	No RCT with renal endpoints
Adiposity, appetite and metabolic inflammation (Section 3.3 and Section 3.4)	GLP-1 RAs; GIP/GLP-1 co-agonists	Kidney-outcome RCT for semaglutide; secondary or post hoc renal analyses for tirzepatide	No dedicated kidney-outcome trial of a co-agonist; weight-independent effects unquantified
Angiotensin II–mediated intraglomerular hypertension (Section 3.1)	ACE inhibitors and ARBs	Long-established RCT evidence in proteinuric CKD	Does not interrupt chymase-derived angiotensin II
Mineralocorticoid receptor activation (Section 3.2)	Finerenone and other non-steroidal MRAs	Kidney and cardiovascular outcome RCTs, confined to T2DM	Application to non-diabetic ORKD is extrapolation; hyperkalaemia risk
Leptin- and chymase-derived aldosterone (Section 3.2.2)	Aldosterone synthase inhibitors	Phase 2 UACR reduction, additive to SGLT2 inhibition	Outcome data awaited
Chymase-driven, aldosterone-independent fibrosis (Section 3.2.3)	Chymase inhibitors	Preclinical only	Species differences limit model validity; no human renal data
Oxidative stress and redox imbalance (Section 3.4.2)	Addressed indirectly via SGLT2 inhibitors, GLP-1 RAs, finerenone	Biomarker-level evidence	Direct antioxidant strategies have not succeeded
Gut dysbiosis and uraemic solutes (Section 3.6)	Dietary fibre; gut-directed strategies	Mechanistic and small clinical studies	No hard renal endpoints

**Table 7:** Therapeutic strategies with renal relevance in ORKD. This table outlines established and emerging therapeutic approaches for ORKD, highlighting their key evidence base, renal signals and relevance to adiposity-driven cardiorenal-metabolic risk.

Intervention/Class	Key Evidence	Renal Signal	Relevance to Obesity-Related CKD	References
SGLT2 inhibitors	CREDENCE, DAPA-CKD, EMPA-KIDNEY	Reduced CKD progression and kidney failure risk across diabetic and non-diabetic CKD populations	Directly counteracts hyperfiltration; modest weight and blood pressure reduction.	Key evidence/renal signal: [24,127,152]. Obesity-CKD relevance: [18,153].
GLP-1 receptor agonists	LEADER and FLOW; semaglutide obesity trials	Albuminuria reduction and reduced kidney/cardiovascular composite endpoints in T2DM with CKD	Substantial weight loss and metabolic-inflammation benefits	Key evidence/renal signal: [25,154,155]. Weight/metabolic relevance: [155,156].
Dual GIP/GLP-1 agonism	Tirzepatide outcome and CKD-focused studies in progress	Favourable albuminuria/eGFR signals in secondary analyses; dedicated CKD data awaited	Potentially powerful weight-loss strategy for obesity-related CKD	Key evidence/renal signal: [157]. Weight-loss relevance: [158]. CKD-specific outcome caveat: dedicated renal outcome data remain limited.
ACE inhibitors/ARBs	RENAAL, IDNT and proteinuric CKD evidence base	Reduced albuminuria and renal endpoint risk in proteinuric kidney disease	Targets adiposity-amplified RAAS and intraglomerular hypertension	Key evidence/renal signal: [59,159,160]. Obesity-RAAS relevance: [125,161].
Finerenone	FIDELIO-DKD and FIGARO-DKD	Reduced CKD progression and cardiovascular events in T2DM with albuminuric CKD	Targets aldosterone/mineralocorticoid receptor-mediated inflammation and fibrosis.	Key evidence/renal signal: [26,27,57]. Mechanistic relevance: [58].
Lifestyle intervention	LOOK AHEAD and CKD exercise studies	Reduced albuminuria and improved cardiometabolic risk factors	Essential background therapy; must avoid sarcopenia and malnutrition	Key evidence/renal signal: [162,163,164]. Sarcopenia/body-composition caution: [49,165].
Metabolic-bariatric surgery	Observational CKD studies and diabetes surgery trials	Proteinuria reduction and improved metabolic drivers; long-term CKD-specific RCT evidence is limited	Most durable weight loss; useful when obesity limits transplantation or CKD control	Key evidence/renal signal: [126,128,166]. ESKD/transplant relevance: [167,168].

This table summarises therapeutic classes rather than providing a full systematic review of trial design. Eligibility, dosing and continuation thresholds should follow current product labelling and CKD guidelines.

**Table 8:** Research priorities for precision nephrology in ORKD. This table identifies key knowledge gaps and proposed research strategies needed to move ORKD care beyond BMI-based assessment toward mechanism-guided precision nephrology.

Priority Area	Unresolved Question	Suggested Approach	References
Adipose depot biology	How do perirenal, renal sinus, visceral and subcutaneous fat differentially affect CKD?	Standardised imaging, adipose transcriptomics and paired renal outcomes	Priority/unresolved question: [17,174,177]. Suggested approach: [50,153].
Phenotype-specific therapy	Which patients benefit most from SGLT2 inhibitors, GLP-1 RAs, finerenone or surgery?	Trials stratified by obesity phenotype, albuminuria, diabetes status and body composition	Priority/unresolved question: [25,57,127,128,152]. Suggested approach: [155,168].
Mechanistic biomarkers	Can lipotoxic, inflammatory and fibrotic injury be distinguished clinically?	Urine/plasma proteomics, metabolomics, single-cell and spatial tissue validation	Priority/unresolved question: [50,131,178]. Suggested approach: [50,131].
Sarcopenic obesity	How should weight loss be prescribed without worsening frailty or muscle loss?	Integrated nutrition, resistance exercise and body composition endpoints	Priority/unresolved question: [49,165,179]. Suggested approach: [165,180].
Transplant access	Can precision risk assessment replace rigid BMI thresholds?	Prospective transplant-centre studies using surgical risk, body composition and functional status	Priority/unresolved question: [167,175,176]. Suggested approach: [168,176].
Ageing with obesity and T2DM	Is the combination a distinct high-risk phenotype rather than three additive exposures?	Cohorts using cystatin C-based eGFR, body composition and functional endpoints; inclusion of frail and sarcopenic participants	See Section 2.3
Perirenal and renal-sinus fat	Does depot thickness predict response to volume-reducing versus anti-inflammatory therapy?	Prospective imaging-stratified trials with paired biomarkers	See Section 3.1.1

These priorities emphasise mechanistic phenotyping, patient-centred outcomes and clinically actionable stratification rather than BMI-only risk assessment.

## Data Availability

No new data were created or analyzed in this study. Data sharing is not applicable to this article.
